# Cardiovascular Exercise Physiology Under Hypoxia, Microgravity, and Heat Stress: A Review with Public Health Implications

**DOI:** 10.3390/ijerph23050594

**Published:** 2026-05-01

**Authors:** Ryan Dumais, Emmett Suckow, Ibrahim Ainab, Francis Zirille, Lindsay M. Forbes, Justin S. Lawley, William K. Cornwell

**Affiliations:** 1 School of Medicine, University of Colorado, Aurora, CO 80045, USA; ryan.dumais@cuanschutz.edu; 2 Department of Medicine-Cardiology, University of Colorado Anschutz Medical Center, 12631 E. 17th Ave, B130, Office 7107, Aurora, CO 80045, USA; 3 Division of Pulmonary, Allergy, and Critical Care Medicine, Department of Medicine, University of Colorado Anschutz Medical Center, Aurora, CO 80045, USA; 4 Clinical Translational Research Center, University of Colorado Anschutz Medical Center, Aurora, CO 80045, USA; 5 Department of Sport Science, University of Innsbruck, 6020 Innsbruck, Austria

**Keywords:** hypoxia, altitude, microgravity, spaceflight, heat stress, heat, VO_2max_, exercise, cardiovascular

## Abstract

**Highlights:**

**Public health relevance—How does this work relate to a public health issue?**

- Population increases in high-altitude environments, the commercialization of spaceflight, and climate change will expose more people to extreme environments.

**Public health significance—Why is this work of significance to public health?**

- A comprehensive understanding of cardiovascular physiology in hypoxia, microgravity, and heat stress environments is critical for the development of evidence-based public health recommendations as the number of people exposed to these conditions increases.

**Public health implications—What are the key implications or messages for practitioners, policy makers and/or researchers in public health?**

- Exposure to hypoxic, microgravity, and heat stress environments negatively impacts the cardiovascular response to exercise and overall exercise capacity.
- Patients and providers must consider the impact of environmental stressors on functional capacity and prepare accordingly. This may be accomplished through acclimatization prior to hypoxic exercise, preservative exercise during exposure to microgravity, and adequate hydration during thermal stress.

**Abstract:**

Aerobic exercise capacity, best quantified by maximal oxygen uptake (VO_2max_), varies between individuals and is dependent on cardiac output (CO) and oxygen uptake in the periphery (a-vO_2_ diff). Environmental stressors like hypoxia, microgravity, and heat negatively impact these parameters, thereby reducing aerobic exercise capacity. However, in response to acute and chronic exposures to these environments, compensatory processes serve to counteract reductions in VO_2max_. In hypoxic environments, reduced oxygen partial pressure (PO_2_) leads to hypoxic pulmonary vasoconstriction (HPV) and a diffusion limitation at the level of the lungs and skeletal muscle, resulting in a reduction in VO_2max_. Microgravity environments reduce VO_2max_ through cardiac and skeletal muscle deconditioning, as well as reductions in plasma volume (PV), resulting in an increase in sympathetic nerve activity through baroreceptor-mediated pathways. In heat stress environments, increases in skin perfusion upon acute exposure hinder exercise performance, whereas compensatory PV expansion mitigates further decreases in VO_2max_. As humans are increasingly exposed to austere environments and environmental extremes, it is critical to understand how these environments impact cardiovascular exercise physiology so that effective strategies and protocols ensuring proper aerobic functioning may be implemented.

## 1. Introduction

While discourse surrounding cardiovascular exercise physiology in extreme environments has often centered around the study of elite athletes, current environmental changes and technological advancements make these topics a public health concern [1]. Not including the millions of people who travel to high-altitude destinations each year, georeferenced data suggest that nearly 82 million people live above 2500 m of elevation, and nearly 15 million people live above 3500 m of elevation [2]. The rapid expansion of commercial spaceflight will expose diverse populations to microgravity environments [3], and the physiological changes that occur during these exposures will continue to provide clinical correlates for medical conditions of public health relevance. Most concerningly, approximately half of the global population is regularly exposed to heat stress, and this proportion will increase over the next several decades as a result of increases in atmospheric temperature [4].

Maintaining aerobic capacity is essential for the preservation of health, functional independence, and productivity in populations exposed to these environmental extremes. Healthy individuals may be limited by reductions in functional capacity while exposed to these stressors. However, those with significant medical conditions, like cardiovascular diseases, may also experience clinical deterioration if unprepared for such exposures [5].

Limitations in aerobic capacity upon exposure to extreme environments are a consequence of perturbations in cardiovascular physiology that are intrinsically optimized for normoxic and normothermic environments under a gravitational field similar to Earth’s (1G = −9.8 m/s^2^). As the body expends energy during aerobic physical activity, oxygen requirements increase to sustain aerobic metabolism. The body has numerous compensatory mechanisms to accomplish this task, with many affecting the cardiovascular system. However, the efficacy of these mechanisms greatly depends on environmental variables such as gravity, temperature, and oxygen partial pressure. Hypoxic environments reduce aerobic exercise capacity primarily through decreased oxygen availability [5,6,7,8]. Microgravity exposure leads to cardiac atrophy, resulting in an adrenergic-mediated rise in heart rate (HR) that limits aerobic capacity [9,10,11,12,13]. Heat stress exposures necessitate increases in skin perfusion for evaporative heat loss, limiting muscle perfusion and aerobic capacity during short-term exposures [14,15,16].

Understanding the cardiovascular adaptations to both exposure and the initiation of exercise in these environments is critical for developing relevant public health guidelines, pre-exposure protocols, and education initiatives for at-risk populations.

In this review, we provide a concise and comprehensive overview of cardiac and pulmonary vascular exercise physiology under normoxic, normothermic, 1G environments. We then explore the physiological adaptations to environmental extremes including microgravity, hypoxia, and thermal stress. Acute physiologic responses and chronic adaptations are discussed as appropriate. For each environment, we use insights from the discussed physiology to promote practical strategies for mitigating functional decline and adverse health events in diverse populations subjected to these exposures.

## 2. Normoxic Exercise

Aerobic capacity is best quantified by the maximal oxygen uptake (VO_2max_) as defined by the Fick equation, represented in Equation (1), where CO is cardiac output and a-vO_2_ diff is the arteriovenous oxygen difference [17]. CO and a-vO_2_ diff are further defined in Equations (2) and (3), where HR is heart rate, SV is stroke volume, CaO_2_ is central arterial oxygen content, and CvO_2_ is central venous oxygen content.

VO_2max_ = CO · a-vO_2_ diff (1)

CO = SV · HR(2)

a-vO_2_ diff = CaO_2_ − CvO_2_ (3)

Based on these equations, one might expect unbounded increases in CO to linearly increase VO_2max_. However, this is not the case. Despite being separate terms in the VO_2max_ equation, CO and a-vO_2_ diff are not independent of one another. This is because oxygen diffusion capacity from the alveoli to the pulmonary arterioles is limited by the speed of blood traversing along the alveoli–arteriole interface. If CO is great enough such that the time the average red blood cell spends at the interface nears the time required for oxygen to diffuse and bind hemoglobin, then oxygen–hemoglobin binding decreases, causing a reduction in the arterial partial pressure of oxygen (PaO_2_) [18,19]. This diffusion limitation generally occurs at very high CO (>32 L/min), although certain disease states limiting the speed of oxygen diffusion may precipitate PaO_2_ reductions at lower CO [19]. The resulting reduction in PaO_2_ reduces the oxygen pressure gradient between capillaries and skeletal muscle tissue, ultimately limiting a-vO_2_ diff during physical activity.

**Figure 1:**
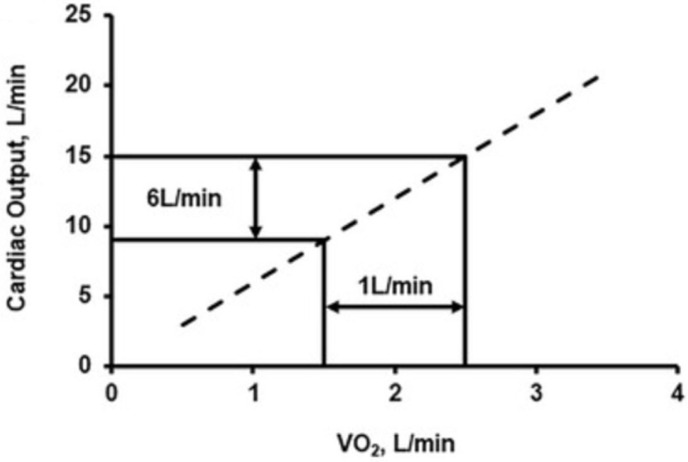
In healthy individuals, VO_2max_ increases by 1 L/min for every 6 L/min increase in CO. Reproduced from Ainab, I.; et al. *Determinants of cardiac output in health and heart failure*. Exp. Physiol. 2025, 110, 637–648 [22]. © 2025 The Authors. Published by Wiley. Distributed under the terms of the Creative Commons Attribution 3.0 License (CC BY 3.0).

Regardless, it is well-established that CO is the primary determinant of VO_2max_ under normal environmental conditions [20,21]. As demonstrated in Figure 1, for every 1 L/min increase in VO_2max_, CO increases by ~6 L/min [22,23]. Additionally, CO can increase from 3-fold to 5-fold during maximal exercise when compared to resting values [24,25]. Thus, CO at maximal oxygen consumption varies significantly between top-tier aerobic athletes and the general population, which corresponds to significant changes in VO_2max_. Part of this difference results from increases in left-ventricular end-diastolic volume and inotropy that develop over time to meet the oxygen demand of intensive aerobic training [26,27,28]. While this cardiac remodeling manifests with SV increases at rest, elite athletes also exhibit greater variations in SV during exercise compared to the general populace, allowing for more than a three-fold increase in SV between rest and maximal exercise [20,29].

The remaining increases in CO that occur during exercise result from elevations in HR. This parameter may also rise significantly between rest and maximum activity, and it is the primary factor driving oxygen delivery during exercise intensities over 50% VO_2max_ since SV generally plateaus beyond this exercise intensity [30].

Despite CO being the primary determinant of VO_2max_ under normal conditions, a-vO_2_ diff also plays a significant role in determining exercise capacity. While a-vO_2_ diff is defined in simplest terms through Equation (3), both CaO_2_ and CvO_2_ are dependent on hemoglobin saturation (SO_2_) and total hemoglobin content (tHb). SO_2_ is further dependent on oxygen partial pressure (PO_2_), internal temperature, and the concentration of hemoglobin allosteric modulators like 2,3-bisphosphoglyceric acid (2,3-BPG), carbon dioxide (CO_2_), and hydrogen ions [31]. Other factors that influence a-vO_2_ diff include skeletal muscle capillary density, mitochondrial density, mitochondrial efficiency, and myoglobin content [32,33]. It is well established that increases in tHb are associated with increases in VO_2max_ and that elite athletes have greater tHb than the general population [34,35,36,37]. Additionally, hemoglobin (Hgb) content in the skeletal muscle circulation is increased during exercise [34,35,36,37]. Both exercised-induced hematopoiesis and adrenergic-mediated constriction of the splanchnic circulation contributes to this increase [38,39,40,41,42,43]. The degree to which tHb influences VO_2max_ varies slightly between studies, with some estimates ranging from a 3 mL/min/kg to 5.8 mL/min/kg change in VO_2max_ per 1 g/kg change in tHb [36,37].

Variations in the efficiency of muscle oxygen extraction and utilization, the latter of which is largely dependent on mitochondrial function and density, also results in significant variations to a-vO_2_ diff as mentioned previously [44]. This principle is demonstrated by studies showing that bed rest deconditioning leads to reductions in skeletal muscle oxygen consumption [44,45]. These reductions are attributable to impairments in mitochondrial assembly and metabolic efficiency secondary to lipotoxic effects from deconditioning [44]. It has also been shown that mitochondrial capacity, described as the maximum rate of total mitochondrial oxygen consumption under optimal conditions, is increased significantly in elite athletes through intensive aerobic training [46].

## 3. Hypoxia

### 3.1. Cardiovascular Exercise Physiology in Hypoxic Environments

Reductions in PaO_2_ that occur in hypoxic (e.g., high altitude) environments pose significant challenges for oxygen consumption and exercise [47]. This challenge is best characterized by the fact that VO_2max_ decreases by 1% for every 100 m increase in elevation above 1500 m [5].

Lung perfusion normally decreases in areas that are poorly oxygenated to maximize oxygen transfer in areas that are well oxygenated. For example, in the setting of impaired oxygen exchange during acute pneumonia, perfusion to the infected area declines in response to reduced ventilation to that area. This unique mechanism, termed hypoxic pulmonary vasoconstriction (HPV), is crucial for maintaining ventilation and perfusion matching [48]. However, in hypoxic environments, all lung areas are poorly oxygenated due to the decreased PO_2_, and as such, there is global pulmonary vasoconstriction. This process not only decreases blood oxygen content directly but also increases pulmonary artery (PA) pressure and right ventricular (RV) afterload [49,50].

**Figure 2:**
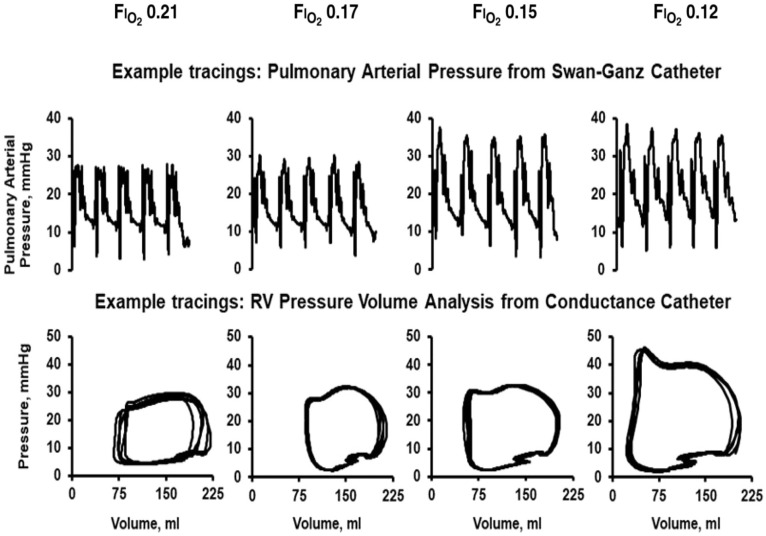
Example hemodynamic tracings demonstrating the response to acute hypoxia. Reproduced with permission from Forbes, L.M.; et al. *Right ventricular response to acute hypoxia among healthy humans*. Am. J. Respir. Crit. Care Med. 2023, 208, 333–336. Published by the American Thoracic Society [49].

In one study demonstrating these changes, ten healthy adults (34 ± 10 years; 3 women; body mass index (BMI) = 24.3 ± 2.7 kg/m^2^) underwent either Swan–Ganz catheterization (N = 5) or conductance catheterization (N = 5) and were acutely exposed to stepwise reductions in fraction of inspired oxygen (FiO_2_) from 0.21 to 0.12 over a 30 min period (Figure 2). Those subjected to Swan–Ganz catheterization showed an increase in systolic PA pressure from (median [interquartile range]) 18 [14,20] to 25 [25,31] mmHg while diastolic PA pressure remained relatively unchanged, yielding a moderate 17% increase in mean PA pressure following FiO_2_ reduction. Meanwhile, those subjected to conductance catheterization exhibited a mild increase in RV afterload from 0.16 [0.15, 0.17] to 0.18 [0.14, 0.18] mmHg/mL as measured by effective arterial elastance (E_A_). Interestingly, data from the conductance catheterization cohort demonstrated that RV contractility, as measured by end-systolic elastance [E_ES_], modestly increased from 0.21 [0.19, 0.25] to 0.24 [0.20, 0.25] mmHg/mL despite the increase in afterload. As a result, ventricular–arterial coupling (E_Es_/E_A_) was preserved [49].

These data suggest that while HPV increases PA pressure and RV afterload, the healthy RV has adequate contractile reserve such that CO is maintained during acute exposure to hypoxia. The preservation of contractility despite an increase in afterload is achieved through a multifactorial process, including adrenergic stimulation through peripheral chemoreceptors and central pathways involving arterial baroreceptors and output from the medulla oblongata [51].

Starling forces also contribute to the maintenance of RV contractility during exposure to hypoxia. While RV afterload increases in response to hypoxia, the resulting increase in RV pressure augments SV through the Frank-Starling principle, thereby augmenting CO [48]. Over time, these SV-mediated increases in CO decline due to PV reductions that result from prolonged respiratory alkalosis, leading to compensatory bicarbonate excretion through diuresis [52,53,54]. However, both Starling and adrenergic mechanisms work to maintain CO during acute exposure to hypoxia.

**Figure 3:**
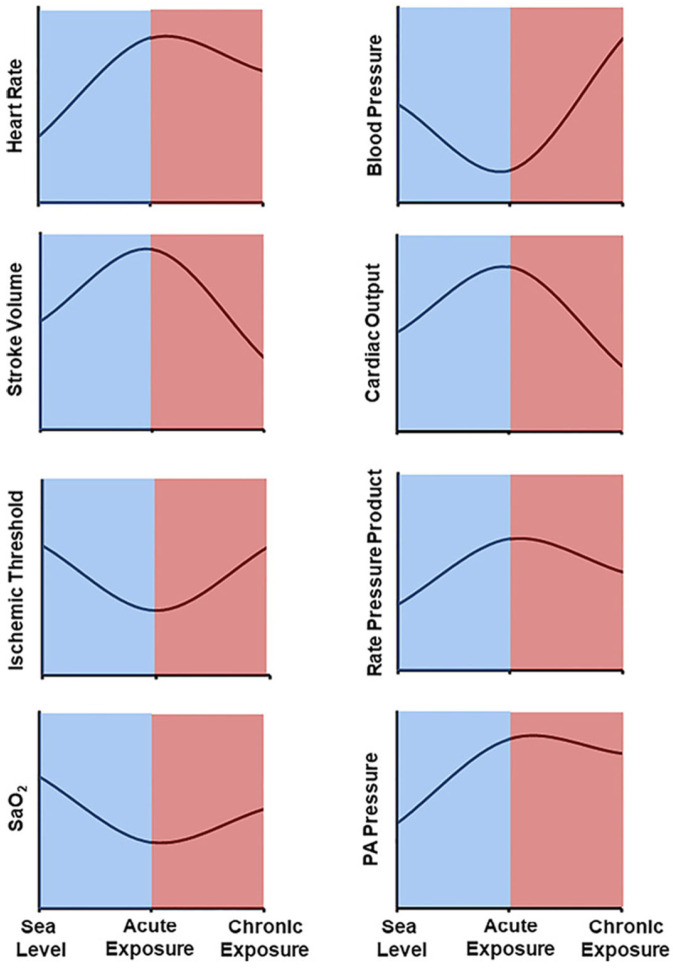
Cardiovascular changes in response to acute (minutes-hours) and chronic (days-weeks) hypoxic exposures relative to normoxic environments. SaO_2_, arterial oxygen saturation. Reproduced with permission from Van Ochten, N.; et al. *The impact of moderate altitude on manifestations of coronary artery disease*. Gerontology 2025, 71, 461–473. Published by Karger Publishers [47].

CO is also influenced by changes in HR that occur in response to hypoxia (Figure 3). In the acute phase of hypoxia, HR increases significantly due to adrenergic activation as previously described. The degree of increase in sympathetic nerve activity is proportional to the severity of hypoxia and is sustained throughout the duration of exposure [51,55]. Thus, while there is a measurable reduction in resting HR over prolonged hypoxic exposures, it remains elevated compared to rest in normoxic conditions [55,56].

Maximal SV during exercise is also reduced in hypoxic environments, resulting in a reduction in maximal CO. In a 2010 study, nine healthy male participants (26.9 ± 1.5 years, BMI = 22.6 ± 0.8 kg/m^2^) completed normoxic (FiO_2_ = 0.21) and hypoxic (FiO_2_ = 0.144) exercise via cycle ergometry while CO was measured using a non-invasive thoracic impedance device. This hemodynamic analysis demonstrated a reduction in SV between the normoxic (163 ± 11 mL/beat, 30.2 ± 1.8 mL/kg/min) and hypoxic (145 ± 11 mL/beat, 26.7 ± 2.1 mL/kg/min) trials [57]. Similar reductions in maximum SV during acute hypoxia have been reproduced in several other studies [58,59,60].

### 3.2. Public Health Implications of Perturbed Exercise Capacity in Hypoxic Environments

The reductions in SV and the increases in PA pressure, RV afterload, and HR that lead to decreased CO and VO_2max_ during exposure to hypoxic environments have profound implications for those living in and visiting high-altitude destinations. For instance, increases in PA pressure and RV afterload upon exposure to hypoxia may predispose those with pre-existing pulmonary hypertension to increased risks of heart failure exacerbations and acute hypoxic respiratory failure [5]. Additionally, elevations in heart rate increase myocardial oxygen demand, predisposing those with heart failure and CAD to develop pulmonary congestion and myocardial infarction respectively [5,61].

For unacclimatized elderly populations, hypoxic environments may also hasten cardiovascular deconditioning through a perceived decrease in energy levels and motivation for physical activity, which may be mediated by diminished cognitive function. While studies investigating the effects of hypoxia on cognitive function have often contradicted each other, a 2025 meta-analysis demonstrated that hypoxia impairs a variety of cognitive domains, including psychomotor skills [62]. Furthermore, it has been demonstrated that impairments in cognitive function impart a deleterious effect on the positive correlation between subjective energy levels and physical activity [63].

These cardiovascular conditions and deteriorations not only reduce quality of life for high-altitude inhabitants, but they may also impair regional economic productivity as unacclimatized populations with cardiovascular diseases may not be able to perform at a high occupational standard. To safeguard vulnerable populations from clinical deterioration in hypoxic environments, it is imperative that clear and practical public health guidelines are adopted and widely distributed. These guidelines should contain both preventative and prospective measures, including physician consultation prior to exposure, portable vital signs monitoring during exposures, consistent accessibility to vital medications, and contingency planning (e.g., route of fastest descent, nearest hospital) in the event of a health emergency [5]. For employers operating in these environments, standard operating procedures (SOPs) such as pre-employment fitness tests, time limits on physical labor, and continuous oximetry monitoring should be implemented to protect those in physically demanding occupations from preventable illness or injury. Finally, local jurisdictions should enact legislation to ensure that SOPs for emergency evacuations are in place and public education initiatives demonstrating risks of exposure to hypoxia are adequate.

While we could not find any widely adopted recommendations for preserving aerobic capacity and reducing the incidence of cardiovascular disease that are specific for high-altitude populations, the epidemiological data surrounding cardiovascular disease in residents living at high altitudes provides key insights into possible suggestions.

It is well established that populations living at high altitudes have a lower incidence of coronary heart disease (CHD) [64,65,66]. In one Swiss study demonstrating this, investigators found that CHD mortality rates decreased (mortality rate per 100,000 person-years [95% CI]) from 289 [275–304] to 242 [193–290] when comparing men residing at <300 m and >1500 m of elevation, respectively [66]. The cause for this reduction in CHD mortality is likely multi-factorial. However, it has been hypothesized that a relative increase in daily physical activity among high-altitude residents, due to the more topographically challenging terrain and hypoxic conditions that high-altitude inhabitants are often required to navigate, may partially explain this trend [67]. This hypothesis is supported by the fact that increases in daily aerobic activity are associated with reductions in CHD mortality risk [68]. Thus, additional recommendations for preserving aerobic capacity and reducing the burden of cardiovascular disease for high-altitude populations, beyond what is currently recommended for the general populace, may not be necessary.

## 4. Microgravity

### 4.1. Cardiovascular Exercise Physiology in Microgravity Environments

**Figure 4:**
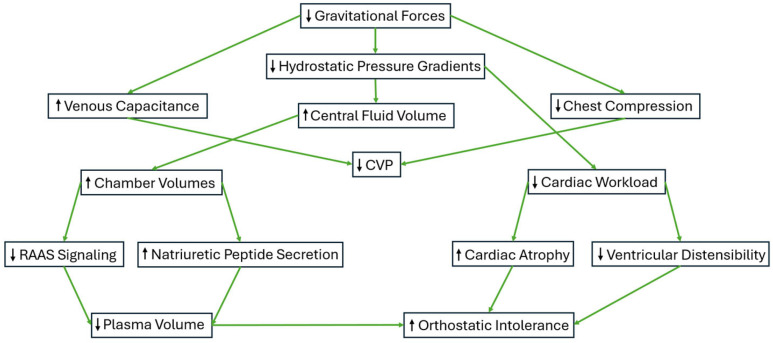
Acute and chronic cardiovascular changes and adaptations in response to microgravity exposure. Black arrows signify the direction of change (↑ = increase, ↓ = decrease). Green arrows signify the factors that act in favor of the outlined changes.

The cardiovascular response to microgravity varies according to the duration of exposure. During the first hours of microgravity exposure, the absence of G-forces eliminates the hydrostatic pressure gradients within the cardiovascular system (Figure 4) resulting in a cephalad fluid shift [69,70]. While these central fluid shifts increase left atrial and left ventricular dimensions, central venous pressure (CVP) decreases [69,71].

In a 1996 landmark study demonstrating these findings, three astronauts were subjected to continuous CVP measurements from pre-launch to acute microgravity exposure via catheterization. This study was the first time astronauts have gone into space with invasive monitoring. The astronauts demonstrated a significant decrease in CVP between the pre-launch seated position (8.4 cmH_2_O) and acute microgravity exposure (2.5 cmH_2_O) even though the left-ventricular end-diastolic internal dimension (LVIDD), measured by echocardiography, increased between supine pre-launch (4.60 cm) and 48 hours post-microgravity exposure (4.97 cm) [69]. This reduction in CVP likely results from reduced chest wall compression of the heart combined with increased venous capacitance secondary to reduced tissue compression against the vasculature [69].

Despite this observed reduction in CVP, it is well-established that atrial and ventricular volumes increase during acute microgravity exposures [69,72,73,74]. In addition to the demonstrated increase in LVIDD in the above study, there was also an increase in the left atrial diameter (LAD) between preflight (3.01 cm) and early inflight (3.25 cm) measurements [69]. These chamber dilations stimulate both arterial baroreceptors and natriuretic peptide secretion, leading to an increase in sympathetic nerve activity and RAAS inhibition [72,73].

Cardiovascular adaptations to microgravity persist throughout the duration of exposure. Specifically, the heart experiences a reduction in workload due to the absence of hydrostatic pressure gradients described earlier, leading to cardiac atrophy. Studies demonstrate that this cardiovascular deconditioning is similar to the deconditioning observed among individuals undergoing bedrest and sedentary aging [75,76,77,78]. The primary consequence of this cardiac atrophy is a reduction in left ventricular end-diastolic volume [10,11,12], and concomitantly, a reduction in ventricular distensibility [79]. Diminished left-ventricular Starling forces impair SV, so to maintain adequate CO, inotropy increases through baroreceptor- and adrenergic-mediated signaling pathways [13].

Cardiovascular deconditioning, which occurs following both microgravity and bedrest exposure, leads to orthostatic intolerance (OI) following re-entry into the atmosphere and re-exposure to an upright gravitational load. In a 1985 study illustrating this phenomenon, five untrained men (45 ± 3 years, BMI = 27.5 ± 4.4 kg/m^2^, VO_2max_ = 30 ± 6.1 mL/kg/min) were subjected to lower body negative pressure (LBNP), before and after 20 hours of bedrest at a −5° tilt [78]. Participants demonstrated a large increase in HR at −40 mmHg of LBNP, which is roughly equivalent to standing upright in 1G, after bedrest (90 ± 18 bpm) compared to baseline conditions (68 ± 12 bpm). Additionally, 4 of 5 participants experienced presyncope at this magnitude of LBNP post-bedrest [78]. These data suggest that cardiovascular deconditioning and OI may even result from short-term (20 hours) bedrest and, by relation, microgravity exposure.

**Figure 5:**
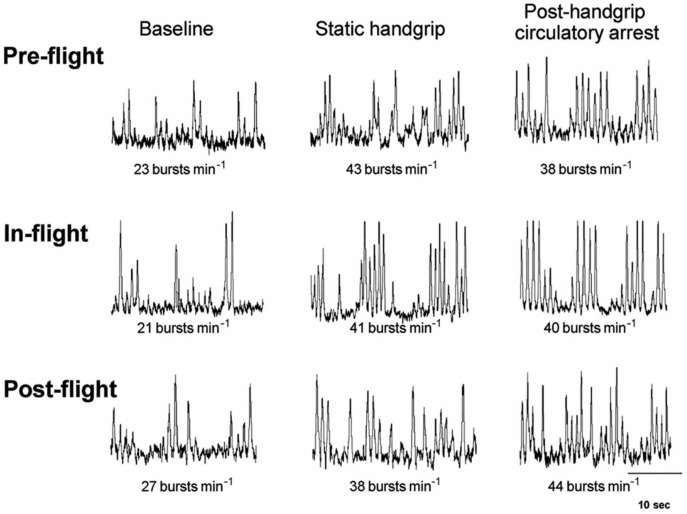
Pre-flight, In-flight, and post-flight MSNA response to static handgrip and post-handgrip circulatory arrest compared baseline for one astronaut. Adapted with permission from Fu, Q.; et al. *Cardiovascular and sympathetic neural responses to handgrip and cold pressor stimuli in humans before, during and after spaceflight*. J. Physiol. 2002, 544, 653–664. Published by Wiley [80].

It is important to note that sympathetic nerve activity is preserved during microgravity exposure. In one study demonstrating this concept, muscle sympathetic nerve activity (MSNA) was measured in five astronauts using microneurography before, during, and after 16 days of spaceflight [80]. As demonstrated in Figure 5, MSNA measured during post-flight resting and post-handgrip circulatory arrest assessments was similar to preflight measurements [80]. These data demonstrate that microgravity does not lead to an acquired dysautonomia. This supports the conclusions of multiple studies demonstrating that, PV reductions and cardiac atrophy are the primary contributors to OI following bedrest [76,81] as opposed to an acquired dysautonomia. Of note, the term “dysautonomia” is inappropriately applied to multiple patient populations suffering from OI and should be used sparingly and appropriately where there is objective evidence to support the diagnosis [82].

While cardiovascular atrophy and OI are important consequences of microgravity, appropriate exercise training can mitigate their severity [83,84]. In a 2023 study, 13 healthy astronauts (4 female, 49.2 ± 4.1 years) undergoing long-duration space missions (155 ± 31 days) were evaluated for changes in left ventricular mass and function via cardiac magnetic resonance imaging (MRI) between preflight and post-flight timepoints and total cardiac work via analytical manipulation of continuous finger blood pressure waveforms between preflight and 15 days prior to re-entry (R-15) measurements. The astronauts performed a minimum of 1.5 h of aerobic and resistance exercise per day during their missions. Results from the study showed that LV mass and SV were preserved between preflight (115 ± 30 g, 93 ± 43 mL) and postflight (118 ± 29 g, 98 ± 39 mL) measurements with appropriate exercise training [83].

Similarly, consistent and intensive exercise training in microgravity can preserve VO_2max_. In a 2014 study, the VO_2max_ of 14 astronauts (5 female, 49 ± 5 years, BMI = 25.2 ± 5.3 kg/m^2^) assigned to long-duration spaceflight missions (164 ± 24 days) was measured at 90 days preflight, flight day 15 (FD-15), and every 30 days thereafter for the duration of their missions using cycle ergometry [9]. The results showed a significant decrease in VO_2max_ between preflight (3.16 ± 0.10 L/min) and FD-15 (2.61 ± 0.10 L/min) measurements but a steady increase in VO_2max_ for the remaining duration of microgravity exposure up to FD-180 (2.82 ± 0.10 L/min) [14].

Data from previous studies regarding VO_2max_ variance during short-duration microgravity exposures is mixed, with two investigations performed during the space shuttle missions demonstrating no change in VO_2max_ [85,86], while a third demonstrated progressive VO_2max_ reductions throughout the mission [87]. These differences are likely a consequence of variations in confounding variables such as cabin atmospheric composition differences and changes in exercise regimens between missions [14].

Regardless, the totality of data demonstrate that VO_2max_ is reduced following exposure to microgravity because of cardiovascular deconditioning and that these reductions in exercise capacity are mitigated by routine exercise training during microgravity exposures.

### 4.2. Public Health Implications of Perturbed Exercise Capacity in Microgravity Environments

With the expansion of commercial spaceflight and the expected progression of space missions transitioning from months to years in duration, understanding the cardiovascular consequences of microgravity exposure is essential for the health and safety of a physiologically diverse cohort of future space travelers. While recent short-duration commercial missions have not produced adverse health outcomes [88], longer duration missions with more medically complex populations have not yet been attempted. This may change soon, however, as upcoming long-duration government-sponsored missions will pave the way for commercialized space travel to follow suit. NASA’s Lunar Exploration Program through Artemis outlines plans for lunar outposts (Artemis Base Camp) and moon-orbiting space stations (The Gateway) [89]. In addition to optimizing lunar life support systems and mission protocols, these projects also aim to simulate components of missions to Mars, enabling future explorations involving significant health and safety challenges for humans in space [89,90].

Commercial spaceflight companies should implement preventative and prospective measures to maintain the functional capacity and aerobic fitness of space travelers, with special considerations for the microgravity-induced cardiovascular changes described above. For longer distance and duration missions, these measures should include pre-flight physical fitness assessments, enforcement of disqualifying medical conditions, adequate and accessible medication supplies during missions, mandatory daily strength and aerobic training, and the implementation of evacuation protocols. While microgravity exposure always carries inherent risk, proactive implementation of these recommendations will reduce the burden of spaceflight-associated adverse medical events as the frequency, duration, and distance of commercial spaceflight missions continues to increase.

Despite the exponential growth of both the spaceflight industry and the number of individuals being exposed to microgravity, most people will never encounter such environments for appreciable periods of time. While microgravity exposures may not pose a direct public health concern in the foreseeable future for this reason, understanding the changes in cardiovascular and exercise physiology that occur in microgravity will provide clinical and therapeutic insights for those who remain on Earth.

As previously discussed, bedrest-induced cardiovascular deconditioning is often used analogously for microgravity-induced cardiovascular deconditioning due to their similar physiological mechanisms and outcomes [91,92]. Thus, preventative and proactive measures to mitigate cardiovascular deconditioning for space travelers may translate clinically to those experiencing deconditioning due to immobilization or decreased functional capacity.

Similarly, insights into cardiovascular physiology during microgravity exposures may contribute to our understanding of conditions like postural orthostatic tachycardia syndrome (POTS), which share many pathophysiologic similarities to microgravity-induced OI, including hypovolemia and cardiovascular deconditioning [93]. Most relevantly, cardiovascular physiological changes that occur during spaceflight share many mechanistic similarities to the poorly understood pathology of long COVID-19. For instance, both long COVID and microgravity exposures result in cardiovascular deconditioning, postural orthostasis, and perturbations in oxygen delivery and extraction leading to decreases in aerobic capacity [78,83,94].

Thus, while microgravity exposure may not currently be a concern of public health magnitude, understanding and further studying the cardiovascular adaptations and limitations that occur in spaceflight environments may provide insights into the mechanisms of and potential therapeutics for pathologies of current public health significance. To ensure that the aerospace industry continues to serve and align with public health interests, it is paramount that leaders in both the private and public aerospace sectors advocate for the design of missions and experiments that translate to issues of public health significance, such as the conditions described above.

## 5. Heat Stress

### 5.1. Cardiovascular Exercise Physiology in Heat Stress Environments

The primary challenge that heat stress environments impose on the cardiovascular system is the requirement of maintaining both tissue perfusion and temperature homeostasis. Increases in internal temperature are primarily mitigated by increases in skin perfusion and eccrine sweat gland secretion [14]. These responses simultaneously release heat through evaporation and cool the body in an efficient process utilizing the relatively high specific heat capacity of water. However, this places strain on the cardiovascular system to accommodate increases in skin perfusion through CO augmentation [95].

Most cardiovascular compensatory processes to heat stress environments occur within a few days of exposure [96,97]. Volume expansion is the primary mechanism through which these adaptations occur, provided that hydration remains adequate. In a 2015 study demonstrating this concept, 7 male athletes (23.3 ± 4.0 years, 24.3 ± 2.8 kg/m^2^, VO_2max_ = 60.4 ± 4.7 mL/kg/min) were subjected to 30 minute sauna exposures (87 °C, 11% relative humidity) immediately following standard daily exercise training sessions for 10 consecutive days. On each of these mornings, Hgb and hematocrit (Hct) levels were recorded via capillary blood samples. To standardize fluid replenishment across subjects, pre- and post-sauna body weights were measured on each day of the sauna intervention, and participants drank 150% of their body mass change within 6 hours of the post-sauna period to maintain hydration. Hgb and Hct levels were also recorded periodically for 17 days prior to the sauna intervention. The change in PV between the pre-sauna intervention and sauna intervention days was calculated from the measured Hgb and Hct based on Equation (4) [16].

Δ%PV = 100 · Hgb_before_/Hgb_after_ · (1 − Hct_after_)/(1 − Hct_before_) − 100 (4)

Results showed a rapid increase in PV between the pre-sauna intervention days and sauna intervention days, peaking on sauna day 4 (Δ%PV = +17.8, 90% CI [7.4, 29.2]). While PV quickly dropped closer to pre-sauna intervention levels for the remainder of the sauna intervention days following this peak, there was still an appreciable increase in PV during this timeframe [16]. Combined, these data demonstrate that consistent exposure to heat stress environments, with adequate hydration, elicits a significant increase in PV when compared to normothermia.

This increase in volume stress is achieved through a multifactorial process but is primarily related to RAAS activation and sodium reabsorption in sweat [98,99,100,101]. The fluid expansion, combined with the increase in CO, is imperative for temperature regulation, as the increase in skin perfusion promotes heat dissipation through sweat production. The increase in CO with PV expansion is primarily achieved through augmentations in SV. As a result, HR is regulated at a normal level as long as PV is maintained [14,98]. The aforementioned 2015 study demonstrates this concept, as it also measured HR upon wakening and during a 5 minute submaximal (125 W) exercise test during both the pre-sauna intervention and sauna intervention days. There was no difference between exercising HR for the two interventions. Interestingly, there was a slight increase in awakening HR on day 2 (Δ%HR = +8.3, 90% CI [−0.8, 18.2]) followed by an immediate, sustained decrease in awakening HR, nadiring on day 6 (Δ%HR = −10.2, 90% CI [−4.0, −15.9]) of the sauna intervention days [16]. This slight observed reduction in HR aligns with the increase in PV.

As noted, the benefits of PV expansion in heat stress environments are only realized if fluid intake is adequate to sustain it. Dehydration, which is a common sequela of ill-prepared exposure to heat stress environments, leads to a reduction in SV with compensatory increases in HR (to maintain CO) and systemic vascular resistance (to maintain blood pressure) [99,102,103]. However, in cases of severe dehydration, these compensatory processes may be inadequate and lead to an increase in core body temperature and ultimately, heat exhaustion and/or heat stroke.

While there are several compensatory mechanisms contributing to preservation of cardiovascular function during acute hyperthermia, there is nevertheless a reduction in exercise capacity [15,104,105,106]. Similar to hypoxia and microgravity environments, such dampening effects can be mitigated with prolonged exposure and preservative aerobic exercise training within heat stress environments. In a 2010 study, 12 cyclists (2 female, 24 ± 6 years, 22.1 ± 3.9 kg/m^2^, VO_2max_ = 66.9 ± 2.1 mL/kg/min) were subjected to VO_2max_ assessments via cycle ergometry in both hot (38 °C, 30% relative humidity) and cool (13 °C, 30% relative humidity) environments. They then participated in a 10-day heat acclimation protocol involving two 45 minute intervals of cycle ergometry per day at 50% of their individual VO_2max_ in hot (40 °C, 30% relative humidity) conditions. VO_2max_ assessments were performed again within the following week of the heat acclimation period using the same protocol as the pre-acclimation assessments. Results showed a significant decrease in VO_2max_ between the cool (VO_2max_ = 66.9 ± 2.1 mL/kg/min) and hot (VO_2max_ = 55.1 ± 2.4 mL/kg/min) pre-acclimation assessments. After the 10-day heat acclimation period, VO_2max_ during the cool (VO_2max_ = 70.2 ± 2.4 mL/kg/min) and hot (VO_2max_ = 59.6 ± 2.0 mL/kg/min) post-acclimation assessments increased significantly relative to their respective cool and hot pre-acclimation assessment values. Despite this, VO_2max_ during the post-acclimation hot temperature assessments did not reach VO_2max_ values achieved in either the pre-acclimation or post-acclimation cool temperature assessments [104]. These data demonstrate that acute heat stress exposures significantly reduce aerobic exercise capacity, but the reduction can be attenuated by acclimation through both exposure prolongation and adequate exercise training throughout the exposure [15,104]. Furthermore, while increases in VO_2max_ following days-long heat acclimation are significant, this attenuation does not fully compensate for the initial decrease in VO_2max_ experienced upon acute exposure to heat stress environments.

### 5.2. Public Health Implications of Perturbed Exercise Capacity in Heat Stress Environments

The cardiovascular and exercise physiological changes that occur during heat stress exposure are a major public health concern for those living in and traveling to areas with high annual temperatures. Similarly to other extreme environmental exposures, populations with significant medical complications are at the greatest risk of deterioration. Cardiovascular diseases are the leading causes of mortality during extreme heat stress events [107], as those with heart failure do not have the reserve capacity to sufficiently increase CO and those with CAD may experience demand ischemia, progressing to cardiac dysfunction and arrhythmias. Many epidemiological studies demonstrate this association between heat stress and cardiovascular mortality. For instance, one Finish study found that heat waves were associated with a significant increase in cardiovascular mortality (percent increase: 9.9%, 95% CI: 7.7–12.1%) [108], corroborating findings from a similar study in China (relative risk: 1.07, 95% CI: 1.03–1.10) [109].

Heavy diuretic use and insufficient water intake are also significant factors associated with mortality in the setting of heat stress, as they lead to dehydration [107]. With insufficient total body water content, PV expansion cannot occur adequately in the setting of heat stress, leaving HR modulation as the primary cardiovascular mechanism for maintaining CO and temperature homeostasis. Severe dehydration and diuretic use during heat stress exposures can also increase mortality through potassium loss, leading to lethal cardiac arrhythmias [110]. For people with chronic kidney disease (CKD), long-term heat stress exposures may further decrease kidney function [111], leading to potassium retention—another cause of lethal cardiac arrhythmias.

Formulating physiology-based public health recommendations and initiatives for populations living in and traveling to heat stress environments is crucial for the prevention of adverse health events. First, non-acclimatized people with significant medical comorbidities should undergo pre-travel evaluation prior to traveling to areas experiencing high temperatures. Physicians should encourage these patients to track their heart rate during physical activity, track environmental temperatures throughout their exposure, and avoid overexertion. Additionally, patients and their companions should be educated on the early and late signs of heat illness like thirst, fatigue, hyperthermia, and altered mentation. Finally, these populations should prepare contingency plans that include evacuation, shelter, and rapidly accessible medical care in cases of functional or physiological deterioration. Finally, all people with access to water should hydrate frequently and keep water on their person both before and during physical activity in heat stress conditions.

From an advocacy perspective, one of the most important avenues for population health experts to target with regard to heat stress is regulation of the occupational sector. It is well established that physical labor under heat stress not only impedes worker productivity but also places strain on the cardiovascular system through dehydration and overexertion [112,113,114]. Recommendations surrounding the quantity, fluid type, frequency, and timing of hydration both before and during physical labor in heat stress environments depend on a variety of factors, including ambient temperature, intensity and duration of exertion, and individual factors like body habitus and rate of sweat secretion [115]. While recommendations promoting personalized fluid regimens through measurement of sweat loss may be physiologically ideal in the setting of exercise under heat stress [116], this approach is not practical at the population level for workers. Utilizing subjective thirst as a personalized indicator for hydration is also not optimal since it may lead to underhydration if workers decide to skip or underutilize hydration breaks for reasons of convenience or work culture [115,116]. Thus, global public health groups should encourage occupation-specific legislation to prevent dehydration and other complications of heat stress in the workplace, such as pre-screening physical exams, work limits, hydration breaks, and accessible cooling facilities for laborers working in these environments [117].

## 6. Conclusions

The cardiovascular system elicits numerous adaptive mechanisms to maintain CO and support exercise in response to external and environmental stressors. Exercise capacity is reduced in response to hypoxia in a dose-dependent fashion [5,6,7,8,45,46,47,51], as well as in response to microgravity [9,10,11,12,13] and heat stress [15,104,105,106]. However, these reductions in exercise capacity can be mitigated through acclimatization, adequate hydration, and adherence to an exercise training program.

The conclusions that may be drawn from this review are limited by bias towards the analysis of physically fit individuals and low sample sizes in certain experiments, the latter of which is particularly apparent in our discussion of microgravity. From a public health perspective, more experiments designed with a focus on the general populace, rather than elite athletes, are necessary to draw more accurate conclusions with regard to the effects of extreme environments on cardiovascular physiology and aerobic capacity.

As humankind ventures to more extreme environments, whether through achievement in spaceflight or fault in climate change, it is imperative to understand the cardiovascular adaptations and compensatory mechanisms that affect aerobic fitness in these environments. With this information, we may ensure humanity is physiologically prepared for such extreme environmental challenges through informed public health policy and education.

## Data Availability

No new data were created or analyzed in this study. Data sharing is not applicable to this article.

## Abbreviations

The following abbreviations are used in this manuscript:

CAD	Coronary artery disease
VO_2max_	Maximal oxygen uptake
CO	Cardiac output
a-vO_2_ diff	Arteriovenous oxygen difference
PO_2_	Oxygen partial pressure
HPV	Hypoxic pulmonary vasoconstriction
PV	Plasma volume
HR	Heart rate
SV	Stroke volume
CaO_2_	Central arterial oxygen content
CvO_2_	Central venous oxygen content
PaO_2_	Arterial partial pressure of oxygen
tHb	Total hemoglobin
SO_2_	Oxygen saturation
2,3-BPG	2,3-bisphosphoglyceric acid
CO_2_	Carbon dioxide
Hgb	Hemoglobin
Hct	Hematocrit
RAAS	Renin–angiotensin–aldosterone system
ADH	Antidiuretic hormone
PA	Pulmonary artery
RV	Right ventricle
BMI	Body-mass index
FiO_2_	Fraction of inspired oxygen
E_A_	Effective arterial elastance
E_ES_	End-systolic elastance
E_ES_/E_A_	Ventricular–arterial coupling
SOP	Standard operating procedure
SaO_2_	Arterial oxygen saturation
CHD	Coronary heart disease
CVP	Central venous pressure
LVIDD	Left ventricular end-diastolic internal dimension
LAD	Left atrial diameter
OI	Orthostatic intolerance
LBNP	Lower body negative pressure
MSNA	Muscle sympathetic nerve activity
MRI	Magnetic resonance imaging
R-X	X days prior to atmospheric reentry
FD-X	Flight day X
POTS	Postural orthostatic tachycardia syndrome
CKD	Chronic kidney disease
